# Obituary Notice of Prof. Horace H. Hayden, Being a Valedictory Address to the Graduating Class of the Baltimore College of Dental Surgery

**Published:** 1844-06

**Authors:** Thos. E. Bond

**Affiliations:** Professor of Special Pathology and Therapeutics, in said College.


THE AMERICAN

### JOURNAL OF DENTAL SCIENCE.

VOL. IV.] JUNE, 1844. [No. 4.

### ARTICLE I.

*Obituary Notice of Prof. Horace H. Hayden, being a Valedictory Address to the Graduating Class of the Baltimore College of Dental Surgery.* By THOS. E. BOND, JR., M. D., Professor of Special Pathology and Therapeutics, in said College. February, 1844.

GENTLEMEN:

Your diligence in study has resulted, as diligence mostly does, in complete success. You have passed through your college terms with credit to yourselves and satisfaction to your instructors, and we have met to confer upon you, publicly and solemnly, the most formal testimonials of your fitness to practise the dental art. Upon an occasion so interesting as this, when tender and generous emotions are more than commonly active; when retrospection and anticipation alternately subdue the feelings and elate the heart, it cannot need the badges of mourning which we wear, to remind you that Death has been among us. That vacant chair has solemn meaning. Dr. Hayden is *dead.* A few weeks ago, he was your *instructor;* your hearts will add, he was your *friend.* In years, in knowledge, in devotion to your interests, he was superior to us all; and his absence from this place, this evening, cannot but invest the occasion with much of gloom: cannot but mingle with your gladness much of sorrow. I do not know how this parting hour between pupils and teachers can be better improved, than by setting before you the example of him we mourn, and thus exhibiting to you, in the history of real life, the advantages of those principles and that conduct which he who is dead, and we who survive, have been anxious to recommend to you. Let him who has often taught with words, once more teach by example, and let the eloquence of a long and useful life combine with the solemn recollections of the dead, to impress upon you all-important lessons.

Dr. Horace H. Hayden was born in Windsor, Conn., on the 13th of October, 1768. A friend of his early childhood speaks of him as a most interesting boy. At a very early age he manifested great vivacity and unusual intelligence. He learned to read almost as soon as he did to talk, and at once contracted that fondness for books which was so remarkable in all his subsequent life. Nor was this the mere childish fondness for narrative which leads all little people to number books among their most amusing toys. Little Hayden's reading was of a character which indicated rare precocity of mind.—He not only toyed with books, he carefully and laboriously read them. Such was his industry and systematic application, that at the age of four years he had read the bible regularly through. It is well worthy of remark, in connection with this anecdote, that from early childhood to the day of his death, the bible was his favorite book. Ardent and inquisitive as he was, and multiplied as were the departments of knowledge which he undertook to explore, no study could allure him from the study of God's providence and grace; no volume could fascinate him away from the majestic revelation of the Almighty will. Though from his youth he knew the scriptures, yet, until hoary age, he learned therein, exhibiting a striking exemplification of the fact, that no man neglects the bible because he knows it well. Few men were more strongly tempted to forget the pious instruction given at their mother's knee than Dr. Hayden was. He was, in very early life, thrown into associations which might have tried the integrity of far riper years, yet in the gayety of youth he did not forget the law of that Being whose fear was so early implanted in his breast. In riper years he passed through the fiery trial of scientific scepticism; and though gifted with a bold and inquisitive mind, and devoting himself with ardor to the study of those sciences more particularly usurped by infidel philosophy, he passed through the fire unscathed, and triumphed where thousands fell. Can it be doubted that the mind of this uncommon man was saved from pollution by his early acquaintance with the oracles of God? Was not his escape from the maelstrom of literary atheism, which once drew into its fearful vortex almost all the brilliant minds that came within its sphere, due to the wisdom of that pious mother who directed the powers of his infant mind to the true source of wisdom?

Dr. Hayden did not only read the bible, he studied it. Eagerly availing himself of every aid he could obtain, he examined the sacred page with the same care with which he conducted all his search for truth. The many notes scattered through his bible indicate the critical particularity with which he examined every doubtful passage, and the careful steps by which he essayed to reach the truth. While a boy, he exhibited a remarkable fondness for natural history, and would frequently surprise his friends by the ingenuity and success with which he would discover objects of interest, while rambling through the fields or woods. I need not remind those who knew him how remarkably this propensity was exhibited in his after life. At ten years of age, he began the study of the ancient languages, but from some cause, now unknown, but probably from want of means, he soon abandoned it. As his knowledge of the world was enlarged by intercourse with books, he became more and more desirous to see the things of which he read. But he was not a child of fortune. Like most other men to whom God has given uncommon minds, he was born of obscure and poor parentage; but young Hayden was not to be daunted by the want of facilities of travel. Goldsmith made the tour of Europe without a penny in his pocket, and without the inclination to earn one. Young Hayden had one advantage of the poet,—he was willing to work his passage to foreign lands, and, at the age of fourteen years, importuned his father so earnestly that at length he reluctantly consented to let him go to sea. Accordingly, in a few days he entered a fine new brig, in the very humble capacity of cabin boy, and in her made two voyages to the West Indies. By this time, his desire of travel was so far satisfied as to induce him to decline, for the present, the life of a sailor, and he returned to school and to his books, with the greater zest for his long separation from the pleasures of study. Neither life upon the ocean, nor servitude in the cabin, had damped his ardent love for the study of nature. What time could be spared from the school house and the duties of home, was devoted to rambles, from which he rarely returned without some trophy of his love of nature.

When sixteen years old, he was compelled to leave school, and apply himself seriously to providing for his maintenance. Very reluctantly he submitted to be bound apprentice to a practical architect, whom he served until he arrived at man's estate. Although he was not pleased with this arrangement, which must of course have seriously interfered with his favorite studies, he addressed himself to his new business with characteristic ardor, and soon found sources of abundant pleasure in the study and practice of building. While his hands were employed, his mind was still more actively at work. He read whatever he could find written on architecture, and not content with studying modern works, sought for the oldest, and, to others, the least attractive. It would have been strange if he had not made great progress by so much diligence. Indeed, I am informed that he has left behind him drawings, and writings, on architecture, which are highly creditable to his knowledge of that beautiful art.

Soon after he came of age, he sailed for the West Indies, and sought employment in his business at Point Petre, Gaudaloupe. He succeeded very well, but the periodical fever compelled him to return to the land of his nativity. In the following year, he again visited the West Indies, and again was driven to the hills of Connecticut, by dread of the pestilence that walketh in darkness and wasteth at noonday. This was the last of Mr. Hayden's adventures beyond sea, in quest of fortune. For several years after his return, he continued to pursue his business with great industry, continually aiming to add to his knowledge, and ambitious of distinction in his vocation. His absence from home, and exposure to the enervating influence of tropical suns, and the more baneful effects of tropical habits and manners, had not lessened his love of knowledge, or disinclined him to the laborious exertion necessary to acquire it. His books were still his constant companions, and, as one who knew him well, strongly expresses it, "he found time for every thing but *idleness.*"

Being dissatisfied with his prospects in Connecticut, he visited New York, when in his twenty-fourth year, and sought employment as an architect. Being successful, he concluded to spend the spring and summer in that city, and in the winter returned to Connecticut. But he was not contented to hibernate at ease; while debarred from his ordinary business by the winter's cold, he found employment for his restless mind, in teaching school in the neighborhood of Hartford. In this new vocation he succeeded so well, that he was strongly urged to continue in it; but a circumstance had now occurred which had turned his restless mind in a new direction, and permanently decided his lot in life. While in New York, he had occasion to call upon Mr. Greenwood, an eminent dentist, for some professional aid. While under treatment, the thought suddenly struck him that he would like to be a *dentist*. What he saw or felt in the manipulations of which he was the subject, to excite his admiration, does not appear, but the notion took fast hold of his mind, and happening about this time to meet some heavy losses in New York, he determined at once to throw up his business, which he had acquired with so much labor, and devote himself to dental surgery.

To this end he procured and read the very few books upon this subject which then were accessible, and having satisfied himself, by various essays, that he need not apprehend deficiency of mechanical skill, he bade farewell at once to architecture and to New York, and turned his face southward in quest of a location where he might hope to win fame and fortune. Arriving in this city, forty years ago, a poor man, without friends, and but imperfectly acquainted with the art upon the practice of which he ventured to depend for subsistence and respectability, his situation would have been discouraging indeed, had he not been sustained by his indomitable energy and enterprizing character, above all, by his earliest and best friend, his bible. Hiring a room in a frame house in Fayette street, Mr. Hayden invited dental practice, and, while inviting, sought to *deserve* it.

He was well aware that the art of dentistry as then understood and practised, was rude and but little entitled to be considered scientific. To a man of his quickness of parts and habitual industry, it was a small thing to equal the best operators of his day.

He aimed to do much more; far from being satisfied with having attained to the highest standard of excellence, then afforded by the art, he labored to elevate the employment to a parallel with his own abilities, and make it worthy of the partiality which he had so suddenly and strangely contracted for it. With this view he studied anatomy and medicine with his accustomed ardor, and though he did not then graduate in medicine, he acquired such extensive knowledge in these departments of science, as gained for him the respect and confidence of the medical profession, and fully entitled him to the honorary degrees conferred on him in later life, by the Jefferson College of Philadelphia, and the University of Maryland, each of which Institutions granted, without application on his part, an honorary diploma of Doctor of Medicine. So respectable was his knowledge of surgery considered, that during the attack by the British upon this city during the last war, his services were put in requisition as surgeon, and his kindness and skill were fully employed in caring for the wounded.

Dr. Hayden rose rapidly in public confidence. He became the companion of the most celebrated physicians and medical professors, who from time to time held the highest professional rank in this city. His opinions were listened to with respect, and his suggestions frequently adopted by them, and so high did he rank in professional estimation, that he was invited to read a course of lectures on dentistry to the medical class of the University, an honor never before or since, conferred upon a practitioner of dentistry. He contributed some valuable papers to medical journals, and entered with great zeal upon physiological researches, especially upon an investigation of the uses of the thyroid gland, which though they did not result in any important discovery, displayed great acuteness of mind, and experimental abilities.

Next to the study of physiology and pathology, Dr. Hayden was devoted to geological investigations. The success with which he examined the mineral peculiarities of this vicinity, are well known, and the discovery of the new mineral which bears his name, (Haydenite,) will always keep up the remembrance of his zeal and excellence in that beautiful department of physical science.

Unlike many who have become distinguished in this branch of study, Dr. H. was a firm believer in revealed religion; he had, as I have before remarked, a profound respect for the bible. Nothing would more readily draw from him indignant reprobation, than remarks which seemed derogatory to the majesty of christian truth. In all his geological inquiries, he never lost sight of the great fact, which many seem so ready to forget, that the earth did not make itself, but was made of God; and therefore Dr. H. solved difficulties by an exceedingly simple process, which to others were mountains in their path. An instance of his short-hand method of accounting for unaccountable things is afforded in the reply which he made to the objections of a scientific gentleman, who was in great perplexity to account for the formation of certain springs and water courses. It is strange, said the doctor, that one who believes that a wise Being made the earth, cannot admit that such a Being might exercise as much prudence and management as is evinced by a gardener who ditches his wet ground, and draws off the surplus water.

Having by the force of his own worth, attained distinguished professional consideration, Dr. H. was anxious to see a general improvement effected in the respectability of the dental art. The land was overrun with ignorant empirics who every where imposed upon the public and disgraced the name of dentist. The art was not regarded as a part of medicine. No schools were provided for instruction in it, and no evidence of qualification for practice could be procured; consequently, the public had no means of distinguishing the man of science from the man of brass, who under the name of dentistry, perpetrated mayhem and robbery, maiming and plundering, under color of dental practice.

Under these circumstances it was first necessary to unite the respectable dentists of the country in an association for the purpose of common effort, and mutual encouragement. This was effected by the formation of the American Society of Dental Surgeons, of which Dr. Hayden was chosen President; a journal was also established for the purpose of gathering and diffusing information on all subjects connected with dental surgery. In connexion with these movements it was thought advisable to establish a Dental College, which should consist partly of medical and partly of dental practitioners, and which should afford those instruction in dental surgery, as a part of medical science, and grant diplomas of graduation to any who should be found worthy to receive such certificates.

Dr. H. did not originate these movements. To one younger, and hence more enthusiastic—one of your professors unfortunately absent from us, this honor is, in a great degree, due. But in Dr. H. Dr. Harris found a willing colaborator. At an age when other men are glad to shun labor, and especially to avoid new enterprizes, Dr. H. entered with all the vehemence of his nature into these several schemes.

The amount of labor required of him as a professor was very great, for one who had already numbered threescore years and ten, nor did he avail himself of the indulgence which his age might well have claimed. He was not satisfied with such exertions as would have comported with his strength. He labored diligently and anxiously, too diligently and too anxiously in the discharge of his duty to his pupils. I need not tell you that he did not labor in vain. His lectures were rich in matter. He had read every thing that could throw light upon the healthy and diseased conditions of the dental structures, and, what was of more importance to his pupils, he had been a close observer and independent thinker for fifty years. The aggregate of the vast knowledge thus acquired from the labors of others, and more especially from his own, was comprehended in his lectures. He loved knowledge for its own sake, and studied nature from the force of that instinctive curiosity which is the animating principle of genius. Like Godman, he pursued truth with indefatigable patience, whether his curiosity was excited by the figure of a pebble, the motion of an insect, or the phenomena of disease. The pleasure of the investigation, and the satisfaction of the many questions which his own fertile mind would present, were all the rewards he asked.

The graphic line, once so aptly used to describe a remarkable character of old,

> "Nil actum credens dum quid superesset agendum,"

might well be applied to our departed friend. He reckoned nothing done while any thing remained to be done. Hence, he was ever active, looking upon acquisition as but preparatory to acquirement, and delighting in effort rather than in possession. Until overtaken by fatal illness, Dr. H. continued to practice and to lecture. There is much reason to fear that laborious bodily and mental exertion exhausted his little strength, and accelerated his death. When no longer able to leave his bed, his chief concern seemed to be for his pupils, who, he feared, would suffer serious loss from the interruption of his lessons. Even when delirium ensued, his mind was evidently busy upon his favorite topics. He would call for his class, and, supposing them to be present, would attempt to lecture. The ruling passion was strong in death,—to the last he talked of science.

Gentlemen, your venerable instructor, your friend, is no longer here. God, in his wisdom, has removed him hence.

"The languishing head is at rest,
Its thinking and aching are o'er,
His quiet immovable breast
Is heaved by affliction no more;
His heart is no longer the seat
Of trouble and torturing pain,
It ceases to flutter and beat,
It never shall flutter again."

You will never again look upon his cheerful face and sparkling eye. You will never again hear his voice, discoursing to you of the wonders of organization, the wisdom of God, the pleasures of health, the cure of disease. But wherever you may wander from this scene of his useful life, let his memory go with you,—let it be dear to you,—let it be useful to you. Let his example urge you to exertion; not that you may attain to thorough knowledge of what is now dental science, but that you may add to that knowledge such discoveries and experience of your own as may vastly augment the common stock, and elevate dental surgery to a far more respectable rank than it now holds in the department of medicine.

Above all, let his memory abide with you, as the recollection of a christian man; of one whom *science* only established in the faith, and who loved revelation in the ratio that he was learned in the book of nature. Let it lead you to thorough confidence in that holy volume which only is the book of wisdom, as well as the fountain of consolation, and let it remind you that wherever you are, under whatever circumstances you may be placed, the broad eye of God is fixed upon you, piercing into the depths of your heart, with as much intensity as though no other being trod the earth upon whom that eye could rest.

It is our humble, fervent prayer, that each one of you may so live beneath that searching look, that it may ever be your greatest happiness to know, "*Thou, God, seest me!*"

